# Expression and phylogenetic analysis of the *zic *gene family in the evolution and development of metazoans

**DOI:** 10.1186/2041-9139-1-12

**Published:** 2010-11-05

**Authors:** Michael J Layden, Néva P Meyer, Kevin Pang, Elaine C Seaver, Mark Q Martindale

**Affiliations:** 1 Pacific Biosciences Research Center, Kewalo Marine Laboratory, University of Hawaii, Manoa, 41 Ahui St Honolulu, HI 96813, USA

## Abstract

**Background:**

*zic *genes are members of the *gli/glis/nkl/zic *super-family of C2H2 zinc finger (ZF) transcription factors. Homologs of the *zic *family have been implicated in patterning neural and mesodermal tissues in bilaterians. Prior to this study, the origin of the metazoan *zic *gene family was unknown and expression of *zic *gene homologs during the development of early branching metazoans had not been investigated.

**Results:**

Phylogenetic analyses of novel *zic *candidate genes identified a definitive *zic *homolog in the placozoan *Trichoplax adhaerens*, two *gli/glis/nkl-*like genes in the ctenophore *Mnemiopsis leidyi*, confirmed the presence of three *gli/glis/nkl*-like genes in Porifera, and confirmed the five previously identified *zic *genes in the cnidarian *Nematostella vectensis*. In the cnidarian *N. vectensis*, *zic *homologs are expressed in ectoderm and the gastrodermis (a bifunctional endomesoderm), in presumptive and developing tentacles, and in oral and sensory apical tuft ectoderm. The *Capitella teleta zic *homolog (*Ct-zic*) is detectable in a subset of the developing nervous system, the foregut, and the mesoderm associated with the segmentally repeated chaetae. Lastly, expression of *gli *and *glis *homologs in *Mnemiopsis*. *leidyi *is detected exclusively in neural cells in floor of the apical organ.

**Conclusions:**

Based on our analyses, we propose that the *zic *gene family arose in the common ancestor of the Placozoa, Cnidaria and Bilateria from a *gli/glis/nkl*-like gene and that both ZOC and ZF-NC domains evolved prior to cnidarian-bilaterian divergence. We also conclude that *zic *expression in neural ectoderm and developing neurons is pervasive throughout the Metazoa and likely evolved from neural expression of an ancestral *gli/glis/nkl/zic *gene. *zic *expression in bilaterian mesoderm may be related to the expression in the gastrodermis of a cnidarian-bilaterian common ancestor.

## Background

**Figure 1:**
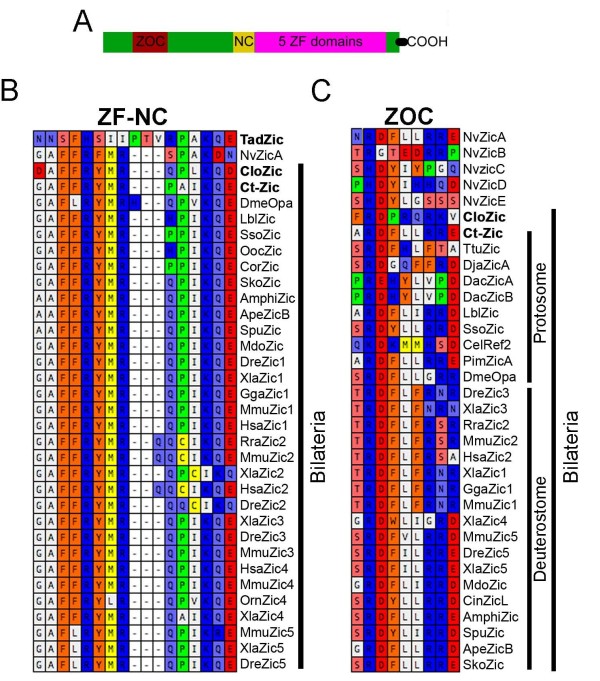
**Alignment of zinc finger (ZF)-nucleocapsid (NC) and Zic 1-3 odd pared conserved (ZOC) domains**. (A) Schematic of *Zic *protein structure. Relative positions of ZOC, ZF-NC, and 5 ZF domains are shown. (B) Alignment of regions immediately N-terminal to the first cysteine of ZF1 for all *Zic *proteins that possess ZF-NC-like consensus sequences. (C) Alignment of ZOC-like sequences for *Zic *proteins that possess a ZOC-like consensus sequence in their amino acid sequence. In both C and D genes identified in this study are in bold.

The *zic *genes form a sub-family of the *gli/glis/nkl/zic *transcription factor super-family, which is characterized by the presence of five tandem C2H2 zinc finger (ZF) DNA binding domains [1-3]. Two key features distinguish the Zic sub-family proteins from the Gli, Glis and Nkl sub-family proteins. Most notably, the number of amino acids between the two cysteine residues in the first C2H2 zinc finger is increased (Additional File 1) [1]. Secondly, many *zic *genes contain two additional domains positioned N-terminal to the ZF domains, the Zic1-3 odd-paired conserved (ZOC) domain and the ZF-NC domain (Figure 1A-C) [1-4]. Function has been assigned to the ZOC domain [4], while the ZF-NC domain has only been identified by sequence conservation.

**Figure 2:**
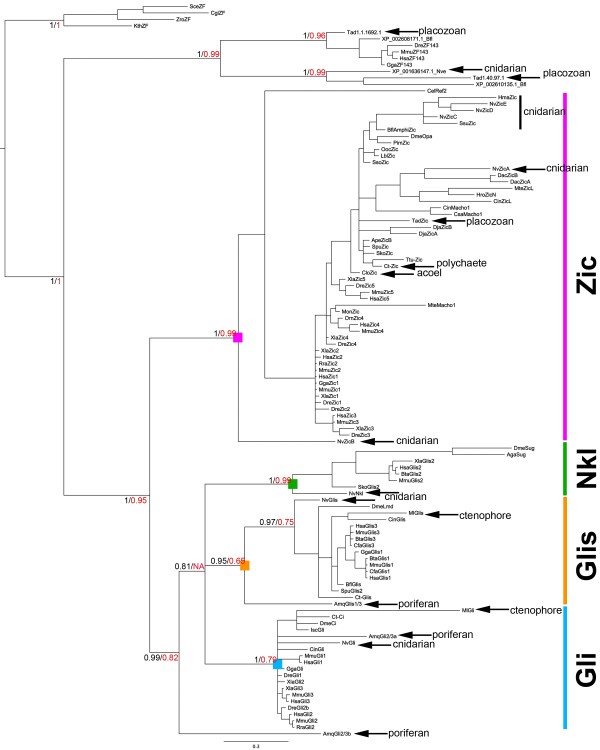
**Gli/Glis/Nkl/Zic super-family phylogeny**. A Bayesian consensus tree generated using the alignment in Additional File 1(see Methods for details). An independent Maximum Likelihood tree generated using PhyML had identical branching pattern at all major nodes. Bayesian posterior probabilities (black) and Maximum Likelihood bootstrap values (red) are shown for identical nodes. Genes identified in this study are demarcated by arrows. The protein families are identified at the node by coloured boxes and corresponding taxa are highlighted by coloured lines. The *Zic *family is demarcated by purple, Nkl by green, Gli by blue and Glis by orange.

Bilaterian and cnidarian *zic *genes likely arose from a single ancestral gene that radiated independently in both lineages. All characterized bilaterian *zic *genes contain a conserved intron between the third and fourth ZF domains that is not present elsewhere in the metazoans [1]. Lineage specific *zic *gene radiation and 100% conservation of this common intron in all bilaterian *zics *support a single urbilaterian *zic *gene. Furthermore, in the basal deuterostome echinoderm *Strongylocentrotus purpuratus *and in most protostome lineages only a single *zic *gene has been identified [1]. An exception is the platyhelminthes lineage, which has been shown to possess two *zic *paralogs (see DjaZicA, B Figure 2) [1]. *zic *gene number expanded via tandem and chromosomal duplication in the chordate lineage from a single ancestral gene in the basal cephalochordate to two in urochordates and five in vertebrates [1-3].

The cnidarians are the only basal metazoan known to possess *zic *gene homologs. A single *zic *gene was identified in polymerase chain reaction (PCR) surveys of *Hydra vulgaris *[5] and the jellyfish *Scolionema suvaense *[1]. *Nematostella vectensis *possesses five *zic *genes, of which four are arranged tandemly on the same genomic scaffold [1], suggesting tandem duplication from a single ancestral gene [1]. Recent sequencing of the *Amphimedon queenslandica *sponge genome identified one putative ancestral *gli/glis/nkl *gene, one *glis*-like gene and one *gli*-like gene [6]. However, no *zic *homolog was identified [6]. *zic *homologs have not been verified in placozoans and ctenophores.

Expression of *zic *homologs during bilaterian development suggests that the urbilaterian *zic *gene was expressed in neural and mesodermal domains. *zic *expression in protostomes (Ecdysozoa and Lophotrochozoa) has been characterized in *Drosophila melanogaster*, *Caenorhabditis elegans *and *Tubifex tubifex*. The fruit fly *D. melanogaster zic *(*odd-paired*; *opa*) is expressed in all ectodermal and mesodermal precursors in the presumptive segmented region of the embryo [7,8]. At later stages *opa *is expressed in the neural ectoderm [9] and in a subset of visceral mesoderm around the midgut [8]. In the nematode *C. elegans*, the *zic *homolog, *ref-2*, is expressed in a subset of neural precursors [10,11]. In the lophotrochozoan oligochaete *T. tubifex*, *Ttuzic *expression is observed in the mesodermal m-blast cells and germ bands, mesoderm associated with chaetal sacs, anterior ectoderm and the developing brain [12]. In summary, protostome *zic *genes are expressed in a sub-set of neural-ectoderm and a sub-set of mesoderm. However, the structures expressing *zic *within a particular germ layer vary between species (subset of mesodermal precursors in *T. tubifex *versus broad mesodermal precursor in *D. melanogaster*). Moreover, expression even within similar tissues (for example, neural) varies in both timing and location (anterior brain in *T. tubifex *versus ventral nerve cord (VNC) in *D. melanogaster*).

In deuterostomes, *zic *expression and function have been best characterized in the chordate lineage. The five vertebrate *zic *gene expression patterns show extensive overlap during development. In the mouse, zebrafish and *Xenopus *embryos, *zic *is expressed in presumptive ectoderm at or just prior to gastrulation [13]. The ectodermal expression resolves to the neural plate and is ultimately restricted to the dorsal neural tube and the lateral boundary of the neural plate where neural crest cells will form [2,3]. In addition, vertebrate *zic *genes are expressed in the dorsal somitic mesoderm and *MmuZic1 *and *MmuZic3 *are expressed in the developing retina [13]. In ascidians, two *zic *genes have been identified (*macho-1 *and *zicL/N*), which display similar patterns among the three species examined [14-18]. *zicL/N *is expressed in presumptive muscle and notochord mesodermal lineages and in neural precursors [14-16]. *macho-1 *mRNA is localized to mesodermal precursor blastomeres [17,18]. The amphioxus *zic *gene (*amphizic*) is detected in the dorsal ectoderm and underlying mesoderm in gastrula, in the mesodermally-derived roof of the anterior archenteron, in the dorsal neural tube and in dorsal-lateral somatic mesoderm in the developing somites [14].

Previously, the only *zic *gene expression pattern to be characterized in early branching metazoans was in the cnidarian *H. vulgaris*. In these animals *zic *(*hyzic*) expression is detected in adult polyps within the dividing i-cells [5], which are stem cells that generate cnidocytes, neurons and other cell types [5]. However, embryonic expression of *zic *gene homologs has yet to be characterized in cnidarians.

In order to better understand the diverse roles of *zic *genes and their potential role in the evolution and development of metazoans, we have investigated the origins and expression of the *zic *gene family in a diverse group of previously unsampled metazoans. Our findings indicate that the *zic *gene family probably arose from a *gli*/*glis/nkl*-like precursor gene in the common ancestor of Placozoa/Cnidaria/Bilateria, as supported by a definitive *zic *ortholog in the *Trichoplax adhaerens *genome and a lack of *zic *homologs in either of the more basally branching Ctenophora or Porifera. Additionally, we present expression patterns for *zic *genes during the development of the cnidarian *N. vectensis*, the lophotrochozoan *C. teleta *and for *gli *and *glis *genes in the ctenophore *M. leidyi*.

## Results

### Zic genes evolved prior to the split between Placozoa and Cnidaria

We identified putative *zic *homologs in early branching metazoans by degenerate PCR and genome searches (see Materials and Methods for details). We searched poriferan, ctenophore, and placozoan genomes because no *zic *sequences had been verified in these metazoan clades. In addition, we searched known fungal genomes and choanoflagellate genomes using Basic Local Alignment Search Tool (BLAST) approaches. In order to identify putative *gli/glis/nkl/zic *genes, sequences were aligned to previously characterized *gli/glis/nkl/zic *genes (see Table 1 for a list of genes and species; Additional File 1 for alignment) and the presence of the five tandem ZF domains was determined. Our search yielded novel candidate *gli/glis/nkl/zic *genes in *T. adhaerens *(three genes), *N. vectensis *(two), *M. leidyi *(two), *C. teleta *(three) and *C. longifissura *(one). However, we were only able to obtain an N-terminal region containing the ZOC, ZF-NC, and the first 2 ZF domains in a putative *zic *from the acoel *Convolutriloba longifissura *(*Clozic*) by rapid amplification of cDNA ends (RACE) PCR (Additional File 1). No choanoflagellete gli/glis/nkl/zic candidate genes were identified in blast searches (data not shown).

**Table 1:** List of genes and abbreviations used in this study.

Phylum	Species	Abbr	Genes used: Accession No./JGI model ID/Sponge ID
Fungi	*Zygosaccharomyces rouxii*	*Zro*	ZroZF [XP_002498870]

	*Saccharomyces cerevisiae*	*Sce*	SceZF [NP_012479]

	*Candida glabrata*	*Cgl*	Cgl [XP_447926]

	*Lancancea thermotolerans*	*Lth*	Lth [XP_002556329]

Ctenophora	*Mnemiopsis leidyi*	*MI*	MIGlis [HM265718], MIGLi [Genbank submission number 1388697]

Porifera	*Amphimedon queenslandica*	*Amq*	AmqGli2/3b [Aqu1.219964], AmqGli2/3a [Aqu1.217717], AmqGlis1/3 [Aqu1.213405]

Placozoa	*Trichoplax adhaerens*	*Tad*	TadZic[XP_002108473], Tad1.40.97.1 [XP_002118240], Tad1.1.692.1 [XP_002107781]

Cnidaria	*Nematostella vectensis*	*Nv*	NvZicA [AB231867], NvZicB [AB231868], NvZicC [AB231868], NvZicD [AB231868], NvZicE [AB231868], NvGli [EU162649], NvGlis [XP_001637240], NvNkl [XP_001636498] Nvpredprot [XP_001636147.1]

	*Scolionema suvaense*	*Ssu*	SsuZic [AB231883 ]

	*Hydra magnipapillata*	*Hma*	HmaZic [XP_002166449 ]

Acoelomorpha	*Convolutriloba longifissura*	*Clo*	CloZic [HM235720]

Platyhelminthes	*Dugesia japonica*	*Dja*	DjaZicA [AB231880], DjaZicB [AB231881]

Dicyemida	*Dicyema acuticephalum*	*Dac*	DacZicA [AB266039 ], DacZicB [AB266038 ]

Annelida	*Tubifex tubifex*	*Ttu*	TtuZic [AB231870 ]

	*Capitella teleta*	*Ct*	Ct-Zic [HM235719], Ct-Glis [e_gw1.3.96.1], Ct-Gli [e_gw1.36.42.1]

Mollusca	*Loligo bleekeri*	*Lbl*	LblZic [AB231874]

	*Octopus ocellatus*	*Ooc*	OocZic [AB231875]

	*Corbicula sp*.	*Cor*	CorZic [BAE94134]

	*Spisula soldissima*	*Sso*	SsoZic [BAE94123]

Nematoda	*Caenorhabditis elegans*	*Cel*	ref2 [AAM55473]

Arthropoda	*Drosophila melanogaster*	*Dme*	opa [NP_524228], ci [NP_524617], Sug [AAS65032], imd [AAF57692]

	*Ixodes scapularis*	*Isc*	IscGli [XP_002435743]

	*Pandinus imperator*	*Pim*	PimZic [AB231877]

	*Anopheles gambiae*	*Aga*	AgaSug [XP_309008] ]

Echinodermata	Strongylocentrotus purpuratus	*Spu*	SpuZic [XP_792929], SpuGlis [XP_798511]

	*Asterina pectinifera*	*Ape*	ApeZic [AB231872 ]

Hemichordata	*Saccoglossus kowaleski*	*Sko*	SkoZic [NP_001158430], SkoGlis2 [XP_002738784]

Chordata	*Ciona intestinalis*	*Cin*	CinZicL [NP_001071853], Cinmacho1 [NP_001027958], CinGli [NP_001071951], CinGlis [NP_001071922]

	*Ciona savignyi*	*Csa*	Csamacho1 [BAB68349]

	*Halocynthia roretzi*	*Hro*	HroZicN [BAC23063]

	*Molgula tectiformis*	*Mte*	MteZicL [BAE54350], Mtemacho1 [BAE54349]

	*Branchiostoma floridae*	*Bfl*	BflZic (aka amphiZic) [CAB96573], BflGlis [XP_002611119], Bflpredprot [XP_002610135.1]

	*Xenopus laevis*	*Xla*	XlaZic4 [BAF36750], XlaZic2 [AAH82436], XlaGli2 [AAD28180], XlaGlis2 [NP_001082092], XlaGli3 [NP_001081440.1] XlaZic3 [NP_001081088.1], XlaZic5 [BAA95699.1]

	*Mus musculus*	*Mmu*	MmuZic5 [NP_075363], MmuZic2 [NP_033600], MmuZic1 [AAH60247], MmuGlis2 [AF325913], MmuGli1 [BAA85004.1], MmuGli2 [NP_001074594], MmuGli3 [NP_032156], MmuGlis1 [AAM93156], MmuGlis3 [ZB131654.1] MmuZic3 [CAM20754.1], MmuZic4 [NP_033602.2], MmuZF143 [EDL16976.1]

	*Rattus rattus*	*Rra*	RraZic2 [NP_001101862], RraGli2 [NP_001100639],

	*Homo sapiens*	*Hsa*	HsaZic2 [AAC96325], HsaGli3 [CAB59315], HsaGlis3 [CAH70655], HsaGlis1 [NP_671726], HsaGli1 [AAM13391.1] HsaGlis2 [NP_115964], HsaZic5 [NP_149123], HsaZic3 [NP_003404], HsaZic1 [NP_003403.2], HsaZic4 [AAH29507.1] HsaZF143 [CAC17610.1]

	*Canis familiaris*	*Cfa*	CfaGlis3 [XP_541295], CfaGlis1 [XP_546702.2]

	*Bos taurus*	*Bta*	BtaGlis1 [XP_615122], BtaGlis2 [DAA15722.1], BtaGlis3 [XP_002689658.1]

	*Monodelphis domestica*	*Mdo*	MdoZic [XP_001376758]

	*Danio rerio*	*Dre*	DreZic4 [NP_001070080], DreGli1 [NP_840081], DreGli2b [NP_001015069], DreZic5 [AAQ67349.1] DreZic1 [NP571008.1], DreZic3 [NP001001950.1], DreZic2 [AAG35717.2], DreZf143 [AA124736.1]

	*Gallus gallus*	*Gga*	GgaZic1 [BAB92091], GgaGlis1 [XP_422485], GgaGli1 [P55878], GgaZF143 [XP_426401.2],

	*Ornithorhynchus anatinus*	*Orn*	OrnZic4 [XP_001507901]

Phylogenetic relationships among candidate genes were determined by performing Bayesian [19] and Maximum Likelihood [20] (Figure 2) analyses using sequences beginning with the first cysteine residue of the first ZF domain (ZF1) through the last histidine residue in the fifth ZF domain (ZF5) (Additional File 1). Our analyses confirmed previously identified cnidarian *zic *genes (*NvzicA-E*) [1] and suggested that the two novel *Nematostella *candidate genes were not *zic *genes but, rather, were members of the *glis *and *nkl *clades. Bayesian and Maximum Likelihood analyses grouped NvGlis with the Glis family (Bayesian posterior probability (PP) = 97, Maximum Likelihood bootstrap value (BS) = 0.75; Figure 2), and grouped NvNkl with the Nkl family (PP = 1, BS = 0.99; Figure 2). Two of the three placozoan *T. adhaerens *candidate genes failed to group within the *gli/glis/nkl/zic *superfamily (see Tad1.1.1692.1 and Tad1.40.97.1; Figure 2), but reciprocal BLAST searches suggested that they were related to ZF143 genes rather than members of the *gli/glis/nkl/zic *gene family. This is supported by phylogenetic analysis for Tad1.1.1692.1 (PP = 1, BS = 0.96; Figure 2). Tad1.40.97.1 groups strongly with the ZF143 (PP = 1, BS = 0.99; Figure 2) genes rather than the *gli/glis/nkl/zic *genes suggesting that it is also not a *gli/glis/nkl/zic *related gene. However the third *T. adhaerens *gene, *Tadzic*, grouped within the *zic *gene sub-family with strong support (PP = 1, BS = 0.99; Figure 2). In the polychaete *C. teleta*, phylogenetic analyses strongly support the assignment of the three candidate genes, *Ct-zic, Ct-gli *and *Ct-glis*, into the *zic, gli and glis *sub-families, respectively (PP = 1, 1, 0.97 and BS = 0.99, 0.79 and 0.75, respectively; Figure 2). In addition, we cloned a partial sequence out of the acoel *C. longifissura, Clozic*, which grouped within the *zic *sub-family (PP = 1, BS = 0.97; Figure 2). The identification of *Tadzic *in Placozoa is the putative earliest metazoan *zic *ortholog identified to date. We conclude that *zic *genes were present in the common ancestor of Placozoa and later branching metazoans.

Two *gli/glis/nkl/zic *sequences were identified in *M. leidyi *despite extensive searches of the *M. leidyi *sequenced genome (50X sequence coverage, Ryan *et al*., manuscript in preparation). One *M. leidyi *sequence, *Mlglis*, grouped with the *glis *sub-family (PP = 0.97, BS = 0.75; Figure 2). The second *M. leidyi *sequence, *Mlgli*, grouped with the *gli *sub-family (PP = 1, BS = 0.79; Figure 2). However, this grouping is based on the presence of only four of the five ZF domains, ZF2-ZF5 (Additional File 1). Attempts to identify the ZF1 domain via RACE PCR and sequence predictions were unsuccessful (data not shown). Thus, we cannot confirm that *Mlgli *contains all five ZFs. However, because it groups so strongly with the *gli *sub-family, we included it in our analysis. We confirmed the reported orthology of the three previously described *A. queenslandica *sequences. We identified an ancestral/sister *gli/glis/nkl *sequence (*Amqgli2/3b*, PP = 0.99, BS = 0.82; Figure 2), one *gli*-like sequence (*Amqgli2/3a*, PP = 1, BS = 0.79), and one *glis*-like sequences (*Amqglis1/3*, PP = 0.95, BS = 0.65). Examination of the spacing between the cysteine residues in the ZF1 peptide sequence is also consistent with *Mlglis*, *Amqgli2/3a, Amqgli2/3b *and *Amqglis1/3 *sequences being more closely related to the *gli/glis/nkl *sub-families than the *zic *gene sub-family (Additional File 1). Degenerate PCR in a second sponge species (*Ephydatia muelleri*) failed to identify a poriferan *zic *sequence (data not shown). We conclude that no definitive *zic *ortholog exists in the ctenophore or poriferan animals analysed, but that both groups contain *gli/glis/nkl*-like homologs in their genomes.

### Clozic and Ct-zic, but not Tadzic, contain ZF-NC domains

As previous studies [1] have suggested that *zic *genes, but not *gli *or *glis *genes, contain a ZF-NC domain, we attempted to identify ZF-NC domains in the newly described *zic *genes *Tadzic, Ct-zic *and *Clozic *as well as in the *gli/glis/nkl *genes. We identified no ZF-NC or ZOC domains outside of the *zic *homologs. The ZF-NC domain is located immediately N-terminal to the ZF1 domain, so we aligned all the sequences N-terminal to the ZF1 domains to identify candidate ZF-NC domains. We identified clear ZF-NC domains in CloZic, Ct-Zic and a partially conserved ZF-NC in TadZic (Figure 1B). The ZF-NC consensus sequence has been previously described as GAF(F/L)RYMRQP-(0-7AA)-IKQE [1]. Ct-Zic and CloZic each have 87% and 93% conserved similarity with this consensus sequence across their respective ZF-NC domains (Figure 1B). However, the TadZic sequence is poorly conserved except for the presence of the four C-terminal amino acids, a conserved proline (Figure 1B), and a conservative arginine to histidine change at position 5 (Figure 1B). While CloZic and Ct-Zic contain definitive ZF-NC domains, it is not clear if the TadZic sequence represents a functional ZF-NC domain.

### A single zic ortholog containing a ZOC domain was shared by Cnidaria and Bilateria

Using non-biased alignment of the N-terminal portions of all Zic proteins assayed, we identified a conserved RDFL-(1-2AA)-RR ZOC consensus sequence. The ZOC domain previously described consensus sequence was (S/T)RDFLxxxR [1,4]. However, our alignment suggests that the RDFL-(1-2AA)-RR consensus sequence is more prevalent throughout the metazoans than the previously described consensus. Using the novel consensus sequence, we identified a ZOC domain in NvZicA,C,D,E, Ct-Zic, and CloZic (Figure 1C). The NvZicA ZOC sequence is 100% identical to the new ZOC consensus sequence. This is particularly important, because ZOC domains have not been previously described in cnidarians [1]. Our finding pushes back the emergence of the ZOC domain to at least the cnidarian-bilaterian common ancestor. The *NvzicC-E *genes possess more weakly conserved putative ZOC domains. However, in the case of *NvzicD*, the amino acid content of the domain HDYIHH, is 100% similar to the RDFL-RR sequence (Figure 1C), suggesting that the *NvzicD *ZOC domain may still be functional. Altering the definition of the ZOC domain results in the inclusion of a number of additional genes and taxa into the ZOC possessing *zic *group. They are the cnidarians (see *Nvzic*A,C,D,E; Figure 1C), the vertebrate Zic4 and Zic5 sequences (see MmuZic5, XlaZic4; Figure 1C), nematodes (see CelRef2; Figure 1C), platyhelminthes (see DjaZicA; Figure 1C) and urochodates (see amphiZic; Figure 1C). We did not find a ZOC-like sequence for TadZic. However, due to sequence and annotation errors, it is possible that we have not identified a full length N-terminal sequence and, thus, a ZOC-like domain may be encoded in the full *Tadzic *coding sequence. Using sequence similarity, it appears that putative ZOC domains can be identified in cnidarians, acoels, platyhelminthes, nematodes, annelids, vertebrates, insects and urochordates (Figure 1C). Thus, the earliest described ZOC sequence appears to have arisen in the cnidarian-bilaterian ancestor, rather than the urbilaterian ancestor.

### Nvzic expression during development of the cnidarian sea anemone *N. vectensis*

We investigated the expression pattern of the five *Nvzic *genes during development of the cnidarian *N. vectensis*. *NvzicA and NvzicB *appear to be expressed at very low levels, as we were unable to obtain reproducible expression for both genes, although they are detected by RT-PCR in 24, 48 and 72 hours post fertilization hpf cDNA samples suggesting that they are expressed at low levels beginning just after gastrulation and maintain expression into at least the planula larval stages (data not shown).

**Figure 3:**
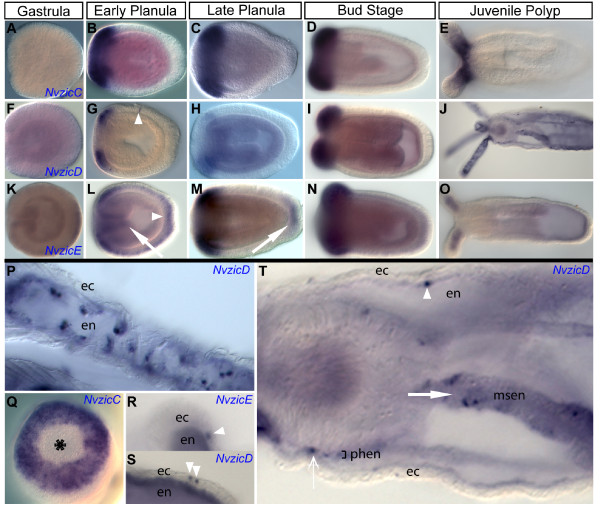
***Nvzic *expression in *Nematostella vectensis***. Expression of *Nematostella NvzicC *(A-E,Q), *NvzicD *(F-J, P, S, T) and *NvzicE *(K-O, R). Late gastrula (A,F,K), early (B,G,L) and late (C,H,M,Q) planula, bud (D,I,N) and polyp (E, J, O, R, S, T) stages are shown. All images are lateral views with oral to the left except Q, which is an oral view with mouth indicated by an asterisk. The endoderm is indicated by en, ectoderm by ec, pharyngeal endoderm by phen and mesentery endoderm by msen. *NvzicC *is expressed in presumptive tentacle and tentacle bud ectoderm (B, C and D) but not in oral ectoderm (Q) in planula stages. The polyp *NvzicC *expression is in the tentacle ectoderm proximal to the polyp body, but not in distal regions (E). *NvzicD *is expressed in the presumptive tentacle ectoderm (G and H) and individual ectodermal cells (G, arrowhead) of the planula. *NvzicD *is also expressed in tentacle buds (I), tentacular endoderm (J and P), pharangeal endoderm (T, open arrow) and in distinct cells in the endodermal component of the directive mesentaries (T, closed arrow) in the polyps. The polyp *NvzicD *expression is occasionally observed in the endodermal cells (T. arrowhead) and the ectodermal cells (S, arrowheads) of the polyp. *NvzicE *expression is expressed in the aboral pole of the gastrula embryo (K, arrow). *NvzicE *is expressed n the oral ectoderm (L, arrow), presumptive tentacle (L and M) and the apical tuft (M, arrow) the planula and tentacle buds (N), tentacle endoderm (O) and occasional individual ectodermal cells in the tentacles (R, arrowhead).

*NvzicC-E *genes all share similar expression patterns. *NvzicC *and *NvzicD *expression is first detectable in the presumptive tentacle ectoderm surrounding the mouth in planula larva (Figure 3B and 3G respectively). There are five notable differences between *NvzicC *and *NvzicD *expression patterns. First, *NvzicD *ectodermal expression is detected only in the presumptive tentacle domains (Figure 3G) and not in the intervening intertentacular ectodermal domains (compare Figure 3G and 3H with 3B and 3C), while *NvzicC *is expressed in a ring encompassing both the presumptive tentacle and intertentacular domains (Figure 3B, C and 3Q). In addition to *NvzicC *ectodermal expression, there might also be expression in the underlying endoderm. The second difference is that, by late planula stages, *NvzicD *expression is down regulated in the presumptive tentacle region (Figure 3H), while *NvzicC *expression remains robust throughout planula and tentacle bud stages (Figures 3B-D). Interestingly, *NvzicD *expression is up regulated again in the forming and early tentacles of the bud and juvenile polyp stages (Figure 3I and 3J). The third difference is that *NvzicD *expression in the juvenile polyp is detectable in distinct endodermal cells or groups of cells in the tentacles (Figure 3J and 3P), while *Nvzic*C is detectable an ectodermal stripe in the proximal tentacle region (Figure 3E). It is currently unclear which cell types are expressing *NvzicD*. The fourth difference is that *NvzicD *can be observed in individual cells in the polyp body wall ectoderm (Figure 3G and 3S; arrowheads). Lastly, *NvzicD *expression is detectable in distinct cell populations in the endodermal component of the developing mesenteries (Figure 3T; closed arrow) and endoderm surrounding the pharyngeal ectoderm (Figure 3T; open arrow), while *NvzicC *is not.

*NvzicE *expression is first detected during embryonic stages, which is earlier than either *NvzicC *or *NvzicD*. It is weakly expressed throughout the embryonic ectoderm shortly after gastrulation (Figure 3K). At planula stages, weak ubiquitous expression of *NvzicE *is detected throughout the ectoderm but enrichment is observed in oral ectoderm and presumptive tentacle bud domain and at the aboral pole in the apical tuft (Figure 3L; arrow and arrowhead, respectively). By late planula stages *NvzicE *expression is down regulated in the body wall ectoderm but remains detectable in the apical tuft ectoderm (Figure 3M; arrow) and the presumptive tentacle domain (Figure 3M). By the tentacle bud stage, aboral expression of *NvzicE *is no longer detectable (Figure 3N), due to the loss of the apical tuft after settlement. However, expression persists in tentacle endoderm (Figure 3O) and, occasionally, single *NvzicE-*positive cells appear in the tentacle ectoderm in juvenile polyps (Figure 3R). In summary, *NvzicC-E *display similar expression patterns in the developing tentacle endoderm and ectoderm, the endodermal component of the mesenteries and in the oral ectoderm. In addition, *NvzicE *is expressed in the apical tuft ectoderm of the late embryonic stages and in individual cells throughout the body of the developing animal.

### Ct-zic expression during development of the bilaterian lophotrochozoan *C. teleta*

Prior to this study *zic *gene expression had only been investigated in a single lophotrochozoan, the oligochaete *T. tubifex*. Most protostome *zic *expression has been described in the ecdysozoans. Thus, investigating additional lophotrochozoan animals will provide a more complete comparison for understanding the relationship among bilaterian *zic *gene expression patterns.

**Figure 4:**
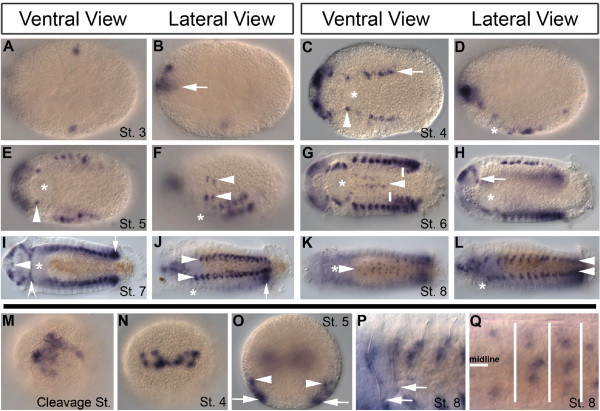
***Capitilla teleta-zic (Ct-zic) *expression in the lophotrochozoan *C. teleta***. Expression of *Ct-zic *at cleavage (M), stage 3 (A and B), stage 4 (C, D and N), stage 5 (E, F and O), stage 6 (G and H), stage 7 (I and, J) and stage 8 (K, L, P and Q). Views are as indicated except that H is a ventral-lateral view; M is an animal view; N and O are anterior views; P is a lateral view; and Q is a ventral view. In all lateral and ventral views the anterior is to the left and the ventral is down in all lateral views. An asterisk (*) indicates the relative mouth position. *Ct-zic *is detectable in micromeres on animal pole (M). *Ct-zic *mesodermal expression is detected in the anterior mesodermal band on the ventral side of stage 3 animals (A and B) and in two bilateral mesoderm domains adjacent to the foregut at stages 4 and 5 (C and E, arrowheads). The mesodermal expression expands around the foregut during stages 6 (G) and 7 (I, notched arrowhead). At stage 5, four lateral mesodermal domains (F, arrowheads) are detected. At stage 7, lateral mesoderm domains form two longitudinal rows along the anterior-posterior (A-P) axis (J, arrowheads). At stage 8, the longitudinal domains are clearly mesoderm associated with chaetae (L, arrowheads, and P), but not the chaetal sac (P, arrows). *Ct-zic *is expressed in presumptive brain ectoderm in all stages (B, arrow, C-L and N). Ventrolateral expression at the lateral edge of ventral neural ectoderm is detected at stage 4 (C, arrow) and stage 5 (F and O, arrows). The lateral neural ectoderm domain is down-regulated in an A-P wave apparent at stage 6 (G, white line demarcates anterior border of expression) but remains expressed in the growth zone (I and J, arrows). *Ct-zic *is also detectable in neurons in the brain (H, arrow) and, beginning at stage 6, in the ventral nerve cord (VNC; G, white arrowhead). VNC expression persists through stage 8 (K, arrowhead, and Q). Medial and lateral ganglions are detected in each segment (Q, which is a higher magnification of L). In Q, the white lines demarcate segmental boundaries.

*Ct-zic *expression in *C. teleta *is first detected during cleavage stages in a subset of micromeres at the animal hemisphere of the blastula (Figure 4M). *Ct-zic *is not detectable in endodermal vegetal macromeres at any stage examined. At later stages, during tissue morphogenesis (stages 3 - 9), *Ct-zic *transcript is detected in the neural ectoderm and mesodermal cell types. Neural ectoderm expression begins at stage 3 in bilaterally symmetric subsets of the anterior neural ectoderm (Figure 4B; arrow), which is also visible from an anterior view at stage 4 (Figure 4N). *Ct-zic *expression is maintained in this anterior neural ectodermal domain throughout development and is present in a sub-region of the developing brain which is clearly visible by stage 6 (Figure 4H; arrow). Previous fate mapping in *C. teleta *has demonstrated that the majority of brain neurons are derived from the anterior neural ectoderm [21,22] and it is likely that *Ct-zic *neurons arise from *Ct-zic *positive domains in the overlying ectoderm.

Neural ectodermal expression of *Ct-zic *is also detected along the lateral edge of the ventral neural ectoderm at stage 4 (Figure 4C and 4O, arrows). The lateral ectodermal *Ct-zic *domain expands toward the posterior of the trunk (Figure 4F), and is then down-regulated in an anterior to posterior wave, visible by late stage 6 (Figure 4G; white lines mark anterior border of expression). From stages 7 - 9, *Ct-zic *expression in the lateral ectoderm is maintained in ventral-lateral cells in the growth zone (Figure 4I, arrow), where new segments are forming. Although the exact lateral border of the ventral neural ectoderm, which forms the ventral nerve chord (VNC), has not been mapped, we think that lateral ectodermal *Ct-zic *expression is within the ventral neural ectoderm. In support of this hypothesis, early neurogenic markers (*Ct-ash *and *Ct-neurogenin*; unpublished observations) positionally overlap with *Ct-zic *expression, although expression of the neurogenic markers extends more medially than *Ct-zic *at similar stages. Furthermore, we observe *Ct-zic *in the VNC beginning at stage 6 (Figure 4G, arrowhead). *Ct-zic *positive cells in the VNC are first detected in anterior segments where *Ct-zic *expression is no longer apparent in the ventral-lateral ectoderm (Figure 4G). By stage 8, *Ct-zic *positive cells are positioned medially and laterally within the VNC (Figures 4K and 4Q). Thus, we conclude that *Ct-zic *is expressed at the lateral edge of the presumptive ventral neural ectoderm, in the VNC, and in a ventral-lateral ectodermal domain in the growth zone where new segments are forming.

Mesodermal expression of *Ct-zic *is detectable at stages 3 to 9. Beginning at stage 3, expression is in one or a few cells in an anterior subset of the bilateral mesodermal bands (Figure 4A and 4B). At stages 4 and 5, mesodermal expression is detected in domains lateral to the foregut (Figure 4C and 4E, arrowheads). The expression around the foregut expands during stages 6 - 7 (Figure 4G and 4I) and is in a sub-set of the *Ct-twist *positive visceral mesoderm surrounding the foregut [23] (Figure 4I, notched arrowhead). In addition, *Ct-zic *expression is observed within a subset of *Ct-twist*-positive anterior mesoderm in the head (Figure 4I, arrowhead).

There is additional *Ct-zic *mesodermal expression in four longitudinal stripes (two on each side of the larva) of segmentally-reiterated cell clusters. This expression domain is first visible in a few cells at stage 5 (Figure 4E, 4F and 4O, arrowheads). The number of *Ct-zic*-positive cell clusters increases along the anterior-posterior (A-P) axis as the animal elongates and new segments form (Figure 4J and 4L, arrowheads). The A-P expansion of this pattern is reminiscent of the expansion of *Ttu-zic *expression in chaetal sac associated mesoderm [11]. By stages 7 and 8, when chaetae are present, *Ct-zic *expression can be clearly observed in cells wrapping the chaetal sacs (Figure 4J, 4L and 4P) but not in the chaetal sacs themselves (Figure 4P, arrows), indicating that the *zic*-positive cells are chaetal-sac associated mesoderm. *Ct-zic *expression is maintained in these structures through stage 9. In summary, mesodermal expression of *Ct-zic *is found in the anterior portion of mesoderm bands at stage 3, in the visceral mesoderm surrounding the foregut, in the head mesoderm at the anterior tip of the animal and in mesoderm associated with forming chaetae.

### *Mlglis *and *Mlgli *expression during development of the ctenophore *M. leidyi*

**Figure 5:**
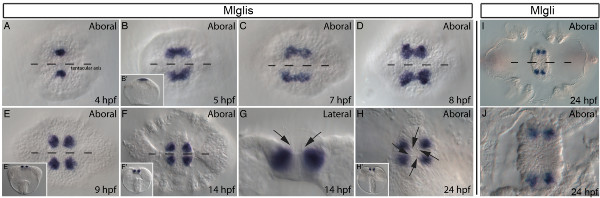
***Mlglis *and *Mlgli *expression during the *Mnemopsis leidyi *development**. Expression of *Mlglis *at 4 high power field (hpf; A), 5hpf (B), 7hpf (C), 8hpf (D), 9hpf (E), 14hpf (F and G) and 24hpf (H) and the expression of *Mlgli *at 24hpf (I and J). The aboral view (A-F, H-J) and the lateral view (G and all insets) are shown. Oral is down in the lateral views and dashed lines indicate the tentacular axis on the aboral views. *Mlglis *expression detected at mid-gastrula in two ectodermal domains flanking aboral pole (A) in the presumptive apical organ domain. Domains expand by late gastrula (B) and assume a dumbbell shape over the next three hours (C and D). By comb row formation, each domain splits resulting in four distinct domains (E), which are maintained through the hatching (F and G) and cydippid (H) stages. The four domains are associated with the balancing cilia at the hatching stages (G arrows) and, in the cydippid larva, optical cross-sections of the balancing cilia are clearly aligned with the centre of each of the four *Mlglis *domains (H arrows). *Mlgli *expression is first detected at 24hpf in four groups of cells along the adesophageal plane of the apical organ (I and J). Unlike *Mlglis*, these cells are not associated with the balancing cilia but are in domains consistent with previously described photoreceptor-like cells [25].

No definitive *zic *homolog was identified in either Porifera or Ctenophora but phylogenetic analyses identified *gli/glis/nkl*-like sequences in both clades (Figure 2). We investigated the expression pattern of *Mlglis *and *Mlgli *during the development of the ctenophore comb jelly *M. leidyi *to gain insight into the evolution of *gli/glis/nkl/zic *expression patterns as the family expanded during metazoan evolution. Both genes showed expression in putative sensory structures in the floor of the apical organ. However, *Mlgli *expression was not detected until very late cydippid larval stages (Figure 5I and 5J) and expression was weaker than the robust expression observed for *Mlglis*.

*Mlglis *expression during *M. leidyi *development is exclusively associated with the apical organ and is not detected in any other tissues. Expression is first detected upon the completion of gastrulation in two opposing groups of cells in the presumptive apical organ ectoderm at the aboral pole of the developing embryo (Figure 5A). Over the next hour of development, expression expands into two arches around the floor plate ectoderm of the apical organ (Figure 5B). The number of cells in each domain expands from approximately four to twenty, though it is not clear if the expansion is due to nascent expression of *Mlglis *in adjacent cells or via cell proliferation of the *Mlglis *expressing cells. Over the next three hours, *Mlglis *domains assume a dumbbell shape and eventually bifurcate (Figures 5C-E), resulting in four distinct domains of expression in the floor plate of the apical organ (Figure 5F). The apical organ contains a statocyst consisting of four groups of cells that give rise to balancing cilia that support the mineral containing lithocytes. The statocyst functions as a balance organ allowing recognition of proper body orientation within the water column. This is achieved by sensing the gravitational pull on the lithocytes perched on the four groups of balancing cilia [24]. *Mlglis *expression is detected in the statocyst cells associated with the balancing cilia (Figure 5G and 5H, arrows) which, based on function, are putative neuronal or neuronal-like cells. In conclusion, *Mlglis *is expressed in the presumptive balancing cilia of the apical organ statocyst beginning shortly after gastrulation and expression is maintained throughout development.

*Mlgli *expression is first detected in 24-hour-old larvae in four groups of putative sensory cells in the floor of the apical organ. *Mlgli-*expressing cells are symmetrically arranged within the floor of the apical organ in the 24 hpf cydippid larva (Figure 5I and 5J). Based on their position, the *Mlgli-*expressing cells and the *Mlglis *expressing cells represent two distinct populations of cells (compare Figure 5J with 5H). The *Mlgli *expressing cells are not associated with the balancing cilia and are positioned further from the centre along the adesophageal plane of the apical organ than the *Mlglis *expressing balancing cilia cells. The domains exhibiting *Mlgli *expression are also associated with putative sensory structures. *Mlgli *expression in the apical organ is reminiscent of previously identified structures, which morphologically resemble photoreceptors by electron microscopy [25]. We conclude that *Mlgli *is expressed in the putative photoreceptive cells in the apical organ and has a distinct pattern from that observed for *Mlglis*.

## Discussion

### The origin of the metazoan zic gene

**Figure 6:**
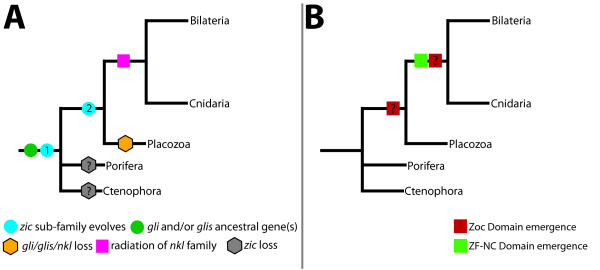
**Model of *zic *gene evolution**. (A) *gli *and *glis *genes were probably present at the base of the metazoan tree (A, green circle). A duplication event occurred either at the base of the Metazoa (A, blue circle 1) or in the last common ancestor of placozoans/cnidarians/bilaterians (A, blue circle 2), which gave rise to the *zic *sub-family. According to this phylogeny placozoans probably lost the *gli/glis/nkl*-like gene(s) (A, orange hexagon). The *nkl *family radiated prior to the cnidarian and bilaterian divergence (A, magenta square). (B) The Zic 1-3 odd paired conserved (ZOC) domain was present in the common ancestor of the bilaterians and cnidarians, but may have emerged in the placozoan/cnidarian/bilaterian common ancestor (B, red boxes with question mark). The ZF-NC domain probably arose in, or just prior to, the cnidarian/bilaterian common ancestor (B, green box).

Understanding the origin of the *zic *gene family is ultimately tied to the understanding of the branching pattern for basal metazoans. There are four basally branching metazoan clades - the cnidarians, placozoans, ctenophores and poriferans. Previous work and our data demonstrate that *zic *genes were present in the placozoan and cnidarian clades, but absent from the current poriferan and ctenophore data sets (Figure 1). Although the phylogenetic relationship between basal metazoans currently is debatable [26-30], the prevailing hypotheses place cnidarians as the sister group to the bilaterians and the divergence of placozoans after the origin of both poriferans and ctenophores [26-29]. Thus, the simplest model is that *zic *family genes diverged once in the common ancestor of the placozoans/cnidarians/bilaterians (Figure 6A, blue circle 2). The alternative hypothesis - that *zic *genes evolved earlier (Figure 6A, blue circle 1) but were lost in more basally branching clades (Figure 6A, grey hexagons) and maintained in placozoans, cnidarians and bilaterians - cannot be ruled out. However, given the conserved expression of *zic *genes in mesodermal and neural tissues, the notion that *zic *genes would be maintained in the placozoans, which have no definitive mesoderm or neural cells, but lost in an ctenophores, which possess muscle cells and a clear nervous system, seems less likely than the notion that *zic *genes evolved prior to placozoan divergence, but after the ctenophore and poriferan radiations. The *T. adhaerens *genome does not possess clear *gli/glis/nkl*-like homologs [31], which may have been lost in conjunction with the loss of these tissues in the placozoan lineage. The simplest model is a single *zic *divergence event in the last common ancestor of the placozoans, cnidarians and bilaterians.

Regardless of when *zic *genes evolved, they probably diverged from a *gli/glis/nkl*-like gene. The basally branching ctenophore and poriferans likely possessed at least two *gli/glis*-like genes, arguing that a *gli/glis/nkl*-like gene, which is lacking in non-metazoans, was present and already duplicated at least once at, or near the time of, the emergence of the earliest metazoans. The presence of the poriferan *Amqgli2/3b *sequence suggests that a third *gli/glis/nkl*-like gene may have existed prior to the divergence of poriferans and ctenophores. However, *Amqgli2/3b *may also represent a lineage specific duplication. As the *gli/glis/nkl *clades represent a single sister group to the *zic *clade, it is unclear whether a *gli*, *glis *or *nkl *gene duplication/divergence would have given rise to the *zic *family. No clear *nkl *genes are present in the basal metazoans, except for the cnidarians, arguing that duplication of a *gli *or *glis*-like gene resulted in the divergence of the *zic *family. However, the existence and phylogenetic position of the *Amqgli2/3b *gene raises concerns about this model. Duplication or divergence of an *Amqgli2/3b*-like gene could have also given rise to the *zic *family. Further resolution of the branching pattern of basal metazoans and sampling for *gli/glis/nkl/zic *genes in ctenophore and poriferan species is required before more a finite hypotheses can be drawn about the evolutionary origin and precursor of the *zic *gene family.

### Two distinct *gli *similar *(glis) *sub-families exist

Though the *glis *and *nkl *gene sub-family names have been used interchangeably, *nkl *should uniquely identify the *glis2/sug *gene family. *glis2 *genes were originally described as neurogenic kruppel like (*nkl*) genes [32], while the glis1/3 genes had been identified as *gli *similar [33]. Our data suggest that the *nkl *and *glis *genes represent two distinct clades. The *glis *clade consists of *glis1*, *glis3 *and the *D. melanogaster lameduck *(*lmd*) homologs (Figure 2), and the *nkl *clade consists of *glis2 *and *D. melanogaster sugarbabe *(*sug*) homologs (Figure 2). This distinction is also supported by a recent investigation of C2H2 ZF genes in amphioxus. The authors of that study identified two distinct strongly supported *glis *clades for *glis2 *and *glis1/3 *genes [34]. We propose that the *nkl *gene sub-family name should be used to uniquely identify the *sug *and *glis2 *genes as a distinct gene sub-family.

### ZOC and ZF-NC domains pre-date emergence of Bilateria

The cnidarian-bilaterian *zic *ortholog contained a ZF-NC-like domain. The first eight amino acids of the published ZF-NC conserved sequence (GAFFRYMR(0-7aa)IKQE) [1] are 86% identical and 100% similar in the putative ZF-NC domain of NvZicA (GAFFRFMR; Figure 1B). In most ZF-NC sequences characterized to date, the arginine residue at position 8 is followed by a QPIKQE sequence (Figure 1B). The corresponding NvZicA sequence (SPAKDN) is 50% similar to the C-terminal portion of the ZF-NC domain (Figure 1B). Lastly, although the first part of the consensus domain is missing in the TadZic sequence, the last four amino acids (AKQE) are 75% identical and 100% similar to the consensus sequence. As no functional mapping has been conducted on the ZF-NC domain and there are strong sequence similarities between the ZF-NC regions of early branching metazoans and bilaterians, it seems likely that presence of a ZF-NC domain may predate the urbilaterian *zic *gene (Figure 6C, green square).

NvZicA-E each contain all, or some of, the reported, and/or proposed, ZOC consensus sequence or have amino acids in similar functional groups to the consensus sequence (Figure 1C). In the case of NvZicE, the actual sequence is not identical to the consensus sequence, but 100% similarity is observed in the five N-terminal amino acids (Figure 1C). In fact, the NvZicE sequence is arguably more similar to the consensus sequence than the oligochaete Ttu-Zic sequence (compare NvZicE and TtuZic; Figure 1C), which has a ZOC domain [1]. Thus, we suggest that the conserved ZOC and ZOC-like domains in the *Nematostella Nvzic *genes supports the inclusion of the ZOC domain in the cnidarian-bilaterian ancestral *zic *gene (Figure 6C, red square). Our results suggest that previously undescribed ZOC domains are present in platyhelminthes, nematode and urochordate *zic *genes. Thus, all sampled bilaterian clades to date now have at least one lineage with a *zic *gene containing a ZOC domain.

ZOC domains have been shown to have two functions in *in vitro *transcription assays. The MmuZic2 ZOC domain has been demonstrated to increase Zic transcription factor activity and to bind the transcription factor Mfa-1 [4]. Currently it is not clear if the ZOC domain increases Zic transcription factor activity via intramolecular interactions, intermolecular interactions or both *in vivo*. Future studies focused on characterizing the activity of cnidarian ZOC sequences are required in order to verify the existence of functional ZOC domains in the cnidarians. Identification and comparative analysis of ZOC interacting proteins in both cnidarian and bilaterian lineages may be useful for determining the molecular relationships of *zic *gene function in metazoan development and evolution.

### Evolution of mesodermal *zic *expression

Mesodermal expression of *zic *genes is present throughout the Bilateria. In *D. melanogaster, opa *is expressed broadly throughout the mesoderm primordia of the segmented region of the embryo [8]. In both lophotrochozoans studied thus far (*T. tubifex *and *C. teleta*) *zic *is expressed in a segmentally reiterated pattern in mesoderm associated with the chaetal sacs [12] (Figure 4) and, in chordates, *zic *expression is observed in a segmented pattern in dorsal domains of forming somites. In addition, within protostomes, *zic *homologs are expressed in visceral mesoderm [surrounding foregut in *C. teleta *(Figure 4) and around a portion of the forming midgut in *D. melanogaster*] [8]. In Cnidaria, we observe *Nvzic *expression in the gastrodermis, a bifunctional endomesodermal tissue, in the forming tentacles, around the pharynx and in the endomesodermal component of the directive mesenteries (Figure 4).

One hypothesis raised by our data is that bilaterian mesodermal *zic *expression is derived, at least in part, from the endomesodermal expression that existed in the common ancestor of the Bilateria and Cnidaria. The expression of *NvzicD *and *NvzicE *in tentacle endomesoderm and *NvzicD *in endomesoderm surrounding the pharynx supports this hypothesis. The tentacles are capable of multiple complex movements, including capture and transport of food to the oral opening due to the high density of myoepithelial cells. It is possible that there are similarities between tentacle endomesoderm and bilaterian musculature. Future work characterizing cell types expressing *NvzicD *and *NvzicE *and comparing the *zic *function in *N. vectensis *endomesoderm will provide an insight to the overall relationship of cnidarian endomesoderm to bilaterian endoderm and mesoderm.

### *zic *expression in neural development

Our data support an ancestral role for *zic *genes as regulators of neural sub-type development. Expression and function of *zic *homologs in neural development is present throughout the Bilateria. However, only in the chordate lineage does *zic *expression and function include the entire neurogenic region in the early embryo. This suggests that, though *zic *genes have a conserved neural role in bilaterian animals, the ancestral role was probably sub-type specification. *Nvzic *genes are expressed in the presumptive and developing tentacle buds in both the endoderm and ectoderm in the cnidarian *N. vectensis *(Figure 3J and 3O and 4E, respectively). The tentacles are highly neuralized structures [35], suggesting that *zic *may be regulating tentacular neurogenesis. In support of this hypothesis, a number of neural genes (*Nvelav, NvanthoRF, Nvmushashi, Nvgcm*) and cross-reactive antibodies against the serotonin and GABA neuropeptides are detected at high levels in the developing tentacle endoderm and ectoderm [35]. In addition, *NvzicE *expression in the forming apical tuft sensory organ (Figure 3M) is consistent with a role in neural development. *zic *expression in the apical tuft of echinoderms has also been described [36]. We describe expression of *Mlglis *and *Mlgli *in the ctenophore *M. leidyi *in neural structures of the developing apical organ (Figure 5). Thus, we suggest that the role of *zic *genes in neural development may have been shared with a *gli*/*glis/nkl/zic *ancestral gene. This is also supported by the observations that *gli*, *glis *and *nkl *genes all have described roles in metazoan neurogenesis [2,3]. Previous studies and our data, taken together, support the hypothesis that one of the ancestral roles of the *zic *gene family was to specify neural sub-types.

### *Zic *homologs may have been co-opted to pattern segmentally reiterated structures along the A-P axis in Bilateria

In the lophotrochozoans *T. tubifex *and *C. teleta*, *zic *is expressed in mesoderm associated with segmentally reiterated chaetae. In amphioxus and vertebrates, *zic *homologs are expressed in the dorsal region of somites. Both somitic expression and mesodermal expression associated with chaetae indicate a role in segmental musculature formation for *zic *homologs in lophotrochozoans and chordates. *C. teleta zic *homologs are also expressed in a reiterated pattern in the ventral nerve cord, reflecting the segmental organization of its body plan (Figure 4). Likewise, *opa *has a role promoting the proneural gene *achaete *expression in each segment during *D. melanogaster *neurogenesis [9]. *opa *mutants were originally identified for their pair-rule phenotype (every other segment disrupted) [37]. However, *opa's *ubiquitous expression throughout the presumptive segmented region is distinct from the typical seven-stripe pair-rule pattern. In addition, *opa *is required for proper expression of the *wg *(wnt) segment polarity gene in all parasegments [7]. Together, these data suggest that *zic *homologs function to pattern segmentally reiterated structures and molecular domains in bilaterians, but are unlikely to function directly in the segmentation process. Rather, the lack of similarity between *zic *expressing reiterated structures across bilaterian lineages suggests that *zic *genes, due to their ancestral roles in neural and mesoderm development in a cnidarian-like ancestor, have been co-opted multiple times downstream of the segmentation programme to pattern various segmentally iterated structures.

## Conclusions

Based on our analysis, we propose that the first metazoan *zic *arose from a *gli/glis/nkl-*like gene prior to the emergence of the Placozoa, but after the divergence of both Ctenophora and Porifera. ZOC and ZF-NC domains were probably present in the ancestral *zic *gene shared by the cnidarian-bilaterian common ancestor. We hypothesize that the metazoan *zic *neural expression, which is present in all animals assayed thus far, may be derived from the expression of an ancestral gene in sensory cells. We also propose that the mesodermal expression of bilaterian *zic *genes may be derived from gastrodermal expression of *zic *homologs in a cnidarian-bilaterian ancestor. Lastly, we suggest that *zic *genes have been co-opted multiple times down stream of segmentation programmes to pattern segmentally reiterated structures in some bilaterian clades. In summary, our results raise an interesting hypothesis about the molecular link between cnidarian endomesoderm and bilaterian mesoderm, and suggest that a role for *zic *genes in neurogenesis may be an ancient metazoan feature.

## Methods

### Identification of candidate *gli/glis/nkl/zic *genes

In order to identify candidate *zic *genes we searched published genomic resources, *Trichoplax adhaerens *http://genome.jgi-psf.org/Triad1/Triad1.home.html, *Nematostella vectensis *http://genome.jgi-psf.org/Nemve1/Nemve1.home.html, *Capitella teleta *http://genome.jgi-psf.org/Capca1/Capca1.home.html, the unpublished genome of the ctenophore *Mnemiopsis leidyi *(NIH), the *Amphimedon queenslandica *http://spongezome.metazome.net/cgi-bin/gbrowse/amphimedon/ and using keyword or TBLASTN and BLASTX searches http://www.ncbi.nlm.nih.gov/BLAST/. We also used previously identified degenerate PCR primers [1] in order to attempt to clone *zic *fragments from the mixed stage embryonic cDNA acoel *Convolutriloba longifissura*, sponge *Ephydatia muelleri *and ctenophore *Mnemiopsis leidyi *using previously described *zic *specific primers [1]. Degenerate fragments were cloned by TA cloning into the pGEM-T Easy vector (Promega, CA, USA) and sequenced (Macrogen, Seoul, Korea). Sequences were used to design nested 5' and 3' RACE primers for each candidate sequence. Full-length sequences were obtained by compiling 5' and 3' RACE sequences. Finally, sequences were confirmed by amplifying the full length sequence from mixed stage embryonic cDNA.

### Phylogenetic analysis

Full length sequences for Gli, Glis and Zic proteins were aligned using the Muscle Alignment [38] web server http://www.ebi.ac.uk/Tools/muscle/index.html. The protein sequences were trimmed using the N-terminal cysteine residue of the ZF1 domain and C-terminal histidine residue of the ZF5 domain to demarcate the five C2H2 ZF domains (Additional File 1). A Bayesian phylogenetic analysis was carried out using MrBayes 3.1.2 [19] with a mixed protein model for 1,000,000 generations sampled every 100 generations with four chains. The Jones model was chosen with a posterior probability of 1. A summary 'consensus' tree was generated in MrBayes using the last 7500 trees (30,000 total trees) representing 750,000 stationary generations. Posterior probabilities were calculated from the 'consensus' tree. A maximum likelihood analysis (using PhyML 3.0 [20]) was also conducted. The Jones model is not present in PhyML 3.0 so the JTT model of evolution (selected via ProtTest [39]) was used with 1000 bootstrap replicates to find the most likely tree: the JTT model is not present in MrBayes. Thus, identical models could not be used for each analysis.

### Alignment of ZOC and ZF-NC domains

The C-terminal domain of full-length alignments derived from Muscle alignment (above) was trimmed from the third amino acid after the first cysteine residue of ZF1. The remaining N-terminal portions of the proteins were then realigned using Muscle and viewed using MacVector version 11.1.1 (MacVector, Inc, NC, USA).

### RNA *in situ *hybridization and image analysis

We performed *in situ *hybridizations, as previously described, for *Mnemiopsis leidyi *[40], *N. vectensis *[32] and *C. teleta *[21]. All images for *M. leidyi *and *N. vectensis *were acquired using a Zeiss Axioskop 2 in conjunction with the Axiocam HRc and Axio vision 4.7 software (Zeiss), Jena, Germany. *C. teleta *images were acquired using a Zeiss Axioskop 2 microscope and a SPOT Flex digital camera (Diagnostic Instruments, Inc, MI, USA) in conjunction with the Spot Advanced version 4.6 software (Diagnostic Instruments, Inc). Additional image processing was done with Helicon Focus software (Helicon Soft Ltd. Kharkov, Ukraine) for some images as indicated in figure legends. Additional images for *N. vectensis *and *M. leidyi *genes shown here are available in the Kahi Kai image database http://www.kahikai.org/.

## Abbreviations

AP: anterior-posterior; BS: bootstrap; *Ct*-*zic *: *Capitella teleta zic *homolog; hpf: hours post fertilization; PCR: polymerase chain reaction; PP: posterior possibility; VNC: ventral nerve cord; ZF: zinc finger; ZOC: *Zic *1-3 oddpaired conserved.

## Competing interests

The authors declare that they have no competing interests.

## Authors' contributions

MJL conceived the study, performed the phylogenetic analysis, performed the *N. vectensis *expression analysis and cloned the *zic *homologs. NPM performed the *C. teleta *expression analysis. KP performed the *M. leidyi *expression analysis and the blast searches of the unpublished *M. leidyi *genome. All authors participated in the drafting and all approved the manuscript.

## Supplementary Material

Additional file 1 **Alignment of *Gli, Glis*, Nkl and *Zic *protein znc finger (ZF) domains**. An alignment of the five tandem C2H2 ZF domains that define the Gli/Glis/Nkl/Zic family is shown. The alignment begins with the first cysteine of ZF1 and ends with last histidine of ZF5. Click here for file
