# Supplementary figures and images for: Expression and phylogenetic analysis of the zic gene family in the evolution and development of metazoans
Source: EvoDevo. 2010 Nov 5;1:12. doi: 10.1186/2041-9139-1-12 (PMC2988786; doi:10.1186/2041-9139-1-12)

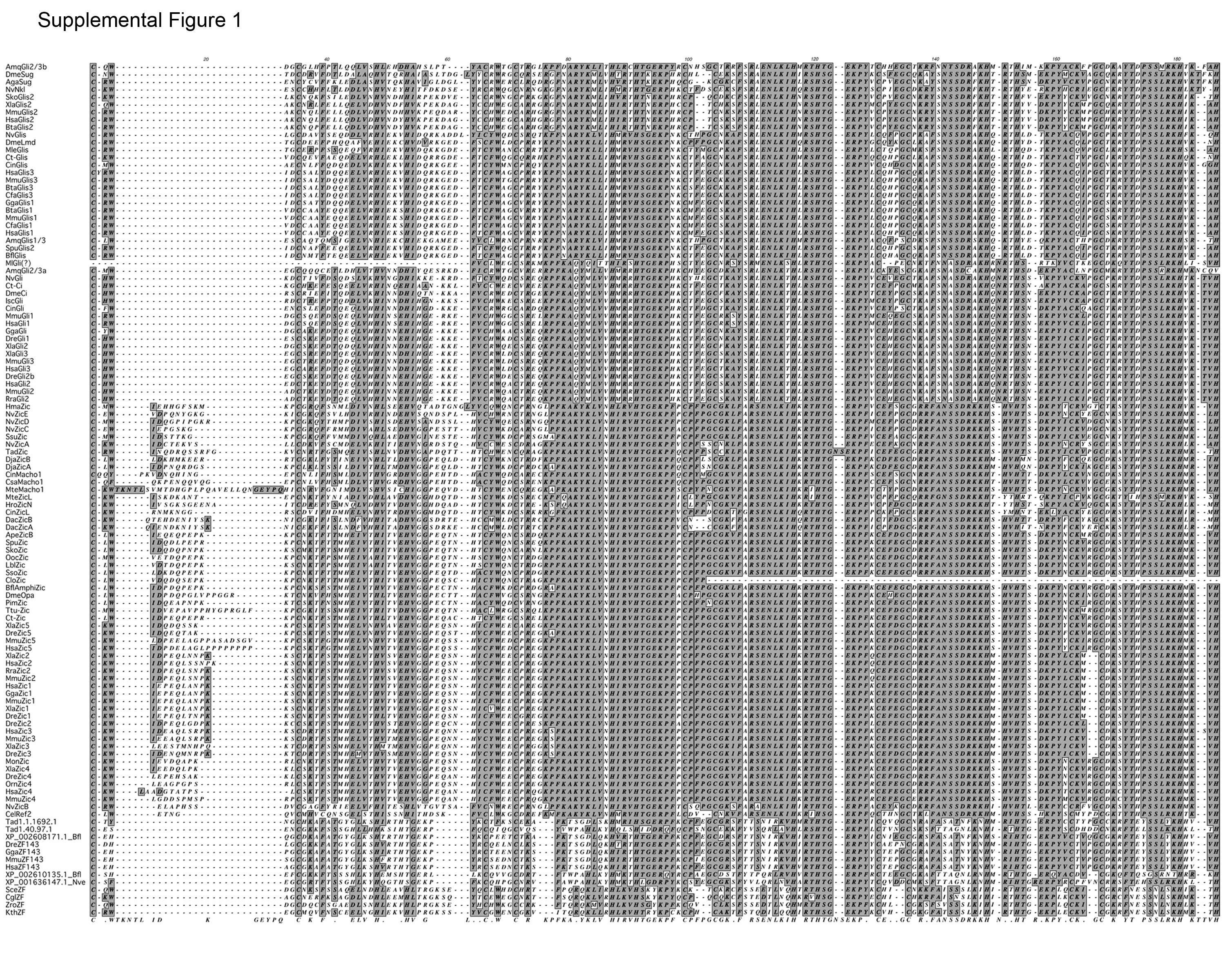

Supplement: Additional file 1 — Alignment of Gli, Glis, Nkl and Zic protein znc finger (ZF) domains. An alignment of the five tandem C2H2 ZF domains that define the Gli/Glis/Nkl/Zic family is shown. The alignment begins with the first cysteine of ZF1 and ends with last histidine of ZF5. [file 2041-9139-1-12-S1.JPEG]
